# An elevated polyclonal free light chain level reflects a strong interferon signature in patients with systemic autoimmune diseases

**DOI:** 10.1016/j.jtauto.2021.100090

**Published:** 2021-03-02

**Authors:** Eléonore Bettacchioli, Christelle Le Gaffric, Margaux Mazeas, Maria Orietta Borghi, Johan Frostegard, Guillermo Barturen, Zuzanna Makowska, Sepideh Babei, Ralf Lesche, PRECISESADS Clinical Consortium, Pier Luigi Meroni, Marta E. Alarcon-Riquelme, Yves Renaudineau

**Affiliations:** a Laboratory of Immunology and Immunotherapy, CHRU Morvan, Brest, France; b Immunorheumatology Research Laboratory, IRCCS Istituto Auxologico Italiano, Milan, Italy; c Section of Immunology and Chronic Disease, Institute of Environmental Medicine, Karolinska Institutet, Stockholm, Sweden; d GENYO, Centre for Genomics and Oncological Research, Pfizer/University of Granada/Andalusian Regional Government, Granada, 18016, Spain; e Bayer Pharma AG, Berlin, Germany; f Drug Discovery, Bayer AG, Berlin, Germany; g Univ Brest, INSERM, LBAI, 29238, Brest Cedex 3, France

**Keywords:** Autoimmune diseases, Free light chains, Autoantibodies, Interferon signature, SAD, systemic autoimmune diseases, RA, rheumatoid arthritis, SLE, systemic lupus erythematosus, SjS, Sjögren's syndrome, Scl, systemic sclerosis, APS, primary antiphospholipid syndrome, UCTD, undetermined connective tissue disease, MCTD, mixed connective tissue disease, HC, healthy controls, IFN, interferon, Ab, autoantibody, FLC, free light chains, RNP, ribonucleoprotein, TNF-R1, tumor necrosis factor receptor 1, CXCL10, C-X-C motif chemokine 10, CCP, cyclic citrulinated peptide, NK, natural killer, TH1, T helper type 1, κ, kappa, λ, lambda, RF, rheumatoid factor, MDA, malondialdehyde, PC, phosphorylcholine, SD, standard deviation, ROC, Receiver Operating Characteristics, AUC, area under the curve, F, female, M, male, VAS, visual analogical scale

## Abstract

High amount of polyclonal free light chains (FLC) are reported in systemic autoimmune diseases (SAD) and we took advantage of the PRECISESADS study to better characterize them. Serum FLC levels were explored in 1979 patients with SAD (RA, SLE, SjS, Scl, APS, UCTD, MCTD) and 614 healthy controls. Information regarding clinical parameters, disease activity, medications, autoantibodies (Ab) and the interferon α and/or γ scores were recorded. Among SAD patients, 28.4% had raised total FLC (from 12% in RA to 30% in SLE and APS) with a normal kappa/lambda ratio. Total FLC levels were significantly higher in SAD with inflammation, active disease in SLE and SjS, and an impaired pulmonary functional capacity in SSc, while independent from kidney impairment, infection, cancer and treatment. Total FLC concentrations were positively correlated among the 10/17 (58.8%) autoantibodies (Ab) tested with anti-RNA binding protein Ab (SSB, SSA-52/60 kDa, Sm, U1-RNP), anti-dsDNA/nucleosome Ab, rheumatoid factor and negatively correlated with complement fractions C3/C4. Finally, examination of interferon (IFN) expression as a potential driver of FLC overexpression was tested showing an elevated level of total FLC among patients with a high IFNα and IFNγ Kirou's score, a strong IFN modular score, and the detection in the sera of B-cell IFN dependent factors, such as TNF-R1/TNFRSF1A and CXCL10/IP10. In conclusion, an elevated level of FLC, in association with a strong IFN signature, defines a subgroup of SAD patients, including those without renal affectation, characterized by increased disease activity, autoreactivity, and complement reduction.

## Highlights

• Polyclonal FLC elevation characterizes patients with SADs.
• Kidney involvement major FLC level elevation.
• Elevated FLC levels are associated with disease activity and autoantibody production.
• A strong interferon signature is associated with elevated FLC levels.

## 1 Introduction

Chronic multisystem autoimmune diseases, referred to as systemic autoimmune diseases (SAD), are diseases of unknown etiology with genetic, epigenetic, sex bias and environmental predispositions [1]. SAD are characterized by a wide range of clinical manifestations and the production of one or more autoantibodies from a large panel of non-organ specific autoantibodies directed at endogenous material, such as cellular and nuclear targets (e.g. anticardiolipin, anti-dsDNA), immunoglobulins (e.g. rheumatoid factor), and post-translationally modified proteins (e.g. CCP). However, at early stages, inflammation and dysregulation of the interferon (IFN) pathway represent common features that characterize SAD and drive the immune response [2]. When activated, usually in concert IFN type I (e.g. IFNα, produced mainly by dendritic cells) and type II (IFNγ, produced by NK cells) promote innate immunity, TH1 phenotype, B cell activation, and plasma cell differentiation. The expression levels of IFN stimulated genes are used to evaluate the IFN activity status in patients.

In SAD, B cell homeostasis is disturbed [[3], [4], [5], [6]] and B cell activation into plasma cells is associated with exaggerated polyclonal synthesis of immunoglobulins together with a slight excess of polyclonal free light chains (FLC) concurrently, with normal kappa(κ)/lambda(λ) ratios. Considered as a waste product of immunoglobulin production, FLC have a serum half-life of 2–6 h, which is 100–200 fold less than immunoglobulin G, and, as such, represent an ideal marker to follow B cell activation as reported in SAD such as SLE, RA, and SjS [7]. Moreover, it is likely that SAD patients present renal impairment in which case an FLC level increase can result not only from overproduction, but also from reduced clearance due to reduced glomerular filtration. Regarding the physiological impact of elevated FLC levels, it has been suggested that FLC are able to inhibit innate immune functions that can contribute to an increase in infections [8], to activate mast cells leading to their degranulation [9], and to promote alternative complement cascade activation and, in turn, nephropathy [10]. An association between elevated FLC levels and an inflammatory burden is also described [11], as well as the capacity of monoclonal FLC to form precursors of amyloid fibrils in amyloidosis [12]. The possible use of 10.13039/100006600FLC as a biomarker in 10.13039/501100009237SAD is further supported by its correlation with disease activity and autoantibody production.

In order to better address the place of FLC in SAD, serum FLC levels were determined in a cross-sectional study including 1979 patients with SAD and 614 healthy controls enrolled in the European's PRECISESAD study. Objectives of the analysis were related to the prevalence of elevated FLC levels in SAD, associations and/or correlations with clinical parameters, disease activity, treatment, autoantibodies and the interplay with the IFN signature in SAD.

## 2 Material and methods

### 2.1 Patients

During the 5-year PRECISESADS project, 1979 SAD patients and 614 healthy controls (HC) were recruited from 18 centers in Europe. According to widely-used classification criteria [[13], [14], [15], [16], [17], [18]], 463 patients with systemic lupus erythematous (SLE), 373 patients with rheumatoid arthritis (RA), 379 patients with Sjögren syndrome (SjS), 397 patients with systemic sclerosis (SSc), 106 patients with primary antiphospholipid syndrome (APS), and 98 patients with mixed connective tissue disease (MCTD) were included for this study. In addition, 163 patients with undetermined connective tissue disease (UTCD) were selected based on the detection of antinuclear antibodies (ANA ≥ 1:160) with or without specific autoantibodies and clinical features of SAD but without fulfilling (i) the aforementioned classification criteria; (ii) 3 criteria of the SLE classification; or (iii) an early systemic sclerosis diagnosis [19].

Fifty four relevant bio-clinical parameters were collected for every patient on the PRECISESADS project (supplementary S2 document), and among them 15 were further analyzed based on their association with FLC overexpression in the SAD population: pleural effusions, venous thrombosis, *sicca* syndrome, gangrene of the fingers, worsening lung function, pulmonary hypertension, pericarditis, erosive arthritis, haemolytic anemia, and systemic hypertension; as well as the following paraclinical markers: inflammation (C-reactive protein > normal (N) or erythrocyte sedimentation rate>30 mm in the absence of current infection), leucopenia (white blood cells <3000 cells/mm3 or lymphocytes <1000/mm3 or neutrophils <1500/mm3), abnormal serum creatinemia (>20% N or increase of >50% versus baseline or a glomerular filtration rate <60 ml/min), proteinuria (excess of proteins in urine >500mg/24 h), and autoimmune hemolytic anemia (hemoglobin < N and haptoglobin < N and positive direct Coombs test). In subanalysis, two subgroups of patients were established to exclude (i) patients with chronic/acute infections and/or a history of cancer; and (ii) patients with renal impairment, based on the presence or past detection of proteinuria and/or abnormal serum creatinine.

Disease activity measures included a 0–100 visual analogue scale (VAS) for global disease activity based on the physician's judgement that was done for all SAD patients. As disease specific activity determination was facultative in the PRECISESADS project, available data include SLE disease activity index (SLEDAI) for 321 SLE patients [20], SjS disease activity index (ESSDAI [21]) for 195 SjS patients and pulmonary functional tests in 204 SSc patients. For pulmonary functional tests, the forced vital capacity (FVC) that measures how much air you can inhale, the diffusing capacity (DLCO) that measures the oxygen capacity that diffuses from the air into the bloodstream^2^, and the ratio FVC/DLCO to predict pulmonary artery hypertension were studied.

The Ethical Review Boards of the 18 participating institutions approved the protocol of the study, which adhered to the standards set by the International Conference on Harmonization and Good Clinical Practice (ICH-GCP), and to the Declaration of Helsinki. The study is registered with number NCT02890121 in ClinicalTrials.gov.

### 2.2 Free light chains (FLC) assay

FLC kappa (FLC-κ) and lambda (FLC-λ) were determined at a single center (Brest, France) using the Freelite® assay through turbidimetry (SpaPlus, The Binding Site, Birmingham, UK). FLC-κ, FLC-λ, total FLC (κ+λ), and κ/λ ratio measures were collected for every patient and healthy controls. For cut-off selection and their comparison, the receiver operating characteristic (ROC) curves were calculated, and the Cohen's kappa coefficient was used for inter-reliability.

### 2.3 Autoantibody assays

A strategy for determining a broad range of autoantibodies in a single center (Brest) was applied, utilizing two distinct panels. The first panel was performed for all samples and included analysis of anti-ENA, anti-CPP2, anti-β2GPI IgG and IgM, anti-CL IgG and IgM, anti-dsDNA, and anti-centromere autoantibodies using the chemiluminescent immunoanalyser IDS-ISYS (Immunodiagnostic, Boldon, UK). With a positive result from the anti-ENA screening test that includes an autoantigen mixture, a second distinct panel was screened for the corresponding autoantigens: Sm, U1-RNP, SSA 52 kDa, SSA 60 kDa, SSB, and anti-Scl70. In all individuals, an ELISA was used for anti-dsDNA-NcX/chromatin detection (Euroimmun, Luebeck, Germany), and turbidimetry to determine rheumatoid factors (RF), complement fractions C3 and C4, and in an unselected subgroup for IgG/A/M isotypes (The Binding Site). The cut-off was chosen according to the manufacturer's instructions except for anti-dsDNA (40 UI/mL) and RF (20 UI/mL) based on results of ROC curve analysis. Anti-malondialdehyde (MDA) and anti-phosphorylcholine (PC) IgM were performed as previously described [22].

### 2.4 Cytokine assays

Cytokines, chemokines and inflammatory mediators were measured in a single center (Milan, Italia) on an unselected subgroup of serum samples from 866 SAD individuals (55 MCTD, 50 APS, 177 RA, 162 SjS, 179 SLE, 180 SSc and 63 UCTD) and 157 healthy controls using the human pre-mixed multi-analyte Luminex assay (R&D Systems, Minneapolis, MN) encompassing 12 cytokines and chemokines involved the inflammatory processes: TNF-R1, CXCL10/IP-10, CXCL13/BLC, GDF-15, IL1R2, CCL8/MCP2, CCL13/MCP4, CCL4/MIP-1b, IL-1RA/TNFRSF1A, CCL17/TARC, MMP8, and FASLG/FAS ligand. The concentration of each mediator was calculated from specific standard curves using the software Bio-Plex Manager v6.0 and expressed as pg/ml.

### 2.5 Expression of interferon-inducible genes and estimation of IFN scores

Total RNA was extracted from an unselected subgroup of whole blood samples from 1275 SAD individuals (70 MCTD, 53 APS, 259 RA, 252 SjS, 294 SLE, 254 SSc and 93 UCTD) and 406 healthy controls, and sequenced on a HiSeq2500 instrument using a HiSeq SBS kit v4 from Illumina (San Diego, CA, USA) after quality controls were run. Reads were then aligned to the *homo sapiens* hg19 reference genome using STAR v2.52b. RSEM v1.2.31 was used for gene expression quantification. For calculation of IFN scores, the expression of IFN inducible genes was used to evaluate the activation of type I (*IFIT1*, *IFI44*, and *PRKR*) and type II (*IRF1*, *GBP1*, and *SERPING1*) IFN pathways according to Kirou's score [23] and by the relative expression of several 5 indicator genes for modules (M)1.2 (*IFI44*, *IFI44L*, *IFIT1*, *IFIT3*, and *MXA*), M3.4 (*ZBP1*, *EIFAK2*, *IFIH1*, *PARP9*, and *GBP4*); and M5.12 (*PSMB9*, *NCOA7*, *TAP1*, *ISG20* and *SP140*) based on Obermoser's report [24]. IFN scores and modules were divided as positive or negative using a threshold defined as the mean of HC + 2 x standard deviation (SD) of HC. According to the number of active IFN modules, patients were stratified into absent (0 module), mild (1 module), moderate (2 modules) or strong (3 modules) IFN signature as previously described [25].

### 2.6 Statistical analysis

Continuous data are described as mean ± SD. Differences between groups were analyzed by multiple one-way ANOVA and the Tukey's test was used for *post hoc* comparison. Receiver Operating Characteristics (ROC) curve analysis was performed to determine the area under the curve (AUC) and the optimal cut-off value was chosen at 95% specificity. Cohen's kappa agreement was calculated for side-by-side comparison, in order to determine the best parameter to keep for the following analysis. The strength of Cohen's kappa was interpreted based on the following definitions: less than chance agreement (<0), slight agreement (0.01–0.20), fair agreement (0.21–0.40), moderate agreement (0.41–0.60), substantial agreement (0.61–0.80), and almost perfect agreement (0.81–0.99). For categorical data (T-test or with Fischer's test, when specified) and non-categorical data (Spearman rank correlation test), the adjusted false discovery rate values (q-values) were calculated and for graphical purpose presented using the –log10 of the q value. Statistical analysis was performed using Prism 7.0 (GraphPad Software Inc, San Diego, CA) or MS-excel 2007 (Microsoft, Redmond WA, USA). Statistical significance was assessed with two-tailed p or q values lower than 0.05.

## 3 Results

### 3.1 Serum FLC in SAD

A total of 2593 individuals were included in the study with 1979 SAD patients and 614 healthy controls (HC), see Table 1. Mean age varied among the SAD subgroups and ranged from 46.1 ± 13.8 years in SLE to 58.6 ± 12.8 years in SjS. The mean age for HC was 46.8 ± 13.2 years. The majority of HC and SAD patients were women (F:M ratio = 3.5 and 6.8, respectively) and of European ancestry (98%). SAD duration was on average 11.9 ± 9.2 years. Using a 0–100 mm VAS based on the physician's judgement to evaluate disease activity, a global mild disease activity was observed from 22.3 ± 19.8 mm, and with important variations between SAD (18.1 ± 24.7 mm in APS versus 30.9 ± 21.2 mm in SSc). Proportions of SAD individuals with infections were 2.2%, with a history of cancer were 7.4%, and with kidney manifestations (abnormal serum creatinine and/or proteinuria per 24 h) were 58% (no)/6.4% (past)/35.6% (present) and 6.8% had nephritis proven by biopsy. The most frequently used therapies were anti-malarial (35.5%), followed by steroids (34.9%), immunosuppressants (34.1%) and biologics (9.2%), mainly in RA for the latter. No cases of hematological malignancies were reported in the PRECISESAD study.

**Table 1:** Clinical characteristics of the studied population.

	Healthy controls	All SAD	RA	SLE	SSc	SjS	MCTD	APS	UCTD
Age (years±SD)	46.8 ± 13.2	52.3 ± 14.3	58.0 ± 12.43	46.1 ± 13.8	58.1 ± 12.9	58.6 ± 12.8	50.5 ± 14.4	48.1 ± 12.2	52.6 ± 14.6
Sex (F:M)	478:138	1724:255	294:79	430:33	335:62	356:23	84:14	76:30	149:14
SAD duration (years±SD)	–	11.9 ± 9.2	12.9 ± 9.9	14.3 ± 9.7	10.9 ± 9.0	10.7 ± 7.8	11.4 ± 9.7	10.0 ± 7.6	8.86 ± 7.74
SAD activity (mm)	–	22.3 ± 19.8	21.1 ± 20.4	18.9 ± 17.6	30.9 ± 21.2	24.5 ± 18.6	24.5 ± 18.95	18.1 ± 24.7	22.1 ± 20.75
No/infection/cancer	–	1782/44/153	349/4/20	412/26/25	352/3/42	338/7/34	92/1/5	85/2/9	145/2/16
No/past/present kidney	–	1148/126/705	241/11/121	217/77/169	266/10/121	202/11/166	70/5/23	54/3/49	98/9/56
FLC-kappa (mg/L)	16.5 ± 14.6	11.7 ± 4.30	13.85 ± 8.28	20.9 ± 22.3	17.1 ± 12.6	21.2 ± 18.7	19.9 ± 14.7	17.2 ± 10.7	13.7 ± 8.39
FLC-lambda (mg/L)	13.7 ± 8.97	10.8 ± 3.77	12.1 ± 6.61	16.7 ± 11.0	15.0 ± 10.4	16.0 ± 11.0	15.9 ± 9.14	14.7 ± 7.27	12.5 ± 6.04
Total FLC (mg/L)	31.2 ± 22.5	23.4 ± 7.32	25.85 ± 13.8	37.5 ± 31.2	32.2 ± 21.2	37.2 ± 27.8	35.7 ± 22.2	31.7 ± 17.1	26.0 ± 13.7
Ratio K/L	1.21 ± 0.51	1.14 ± 0.40	1.21 ± 0.62	1.22 ± 0.54	1.19 ± 0.45	1.32 ± 0.61	1.26 ± 0.45	1.17 ± 0.35	1.10 ± 0.34
Treatment									
Antimalarial	–	703/1979	61/373	325/463	30/397	139/379	45/98	26/106	78/163
Steroids		691/1979	165/373	239/463	99/397	80/379	49/98	8/106	51/163
IS	–	675/1979	283/373	162/463	106/397	60/379	39/98	4/106	23/163
Biologics	–	183/1979	165/373	0/463	10/397	0/379	4/98	0/106	5/163

Abbreviations: SAD: systemic autoimmune disease; RA: rheumatoid arthritis; SLE: systemic lupus erythematosus; SSc: systemic sclerosis; SjS: Sjögren's syndrome; MCTD: mixed connective tissue disease; APS: primary antiphospholipid syndrome; UCTD: undifferentiated connective tissue disease; IS: immunosuppressant; FLC: free light chains; F:M female versus Male; SD: standard deviation.

**Fig. 1:**
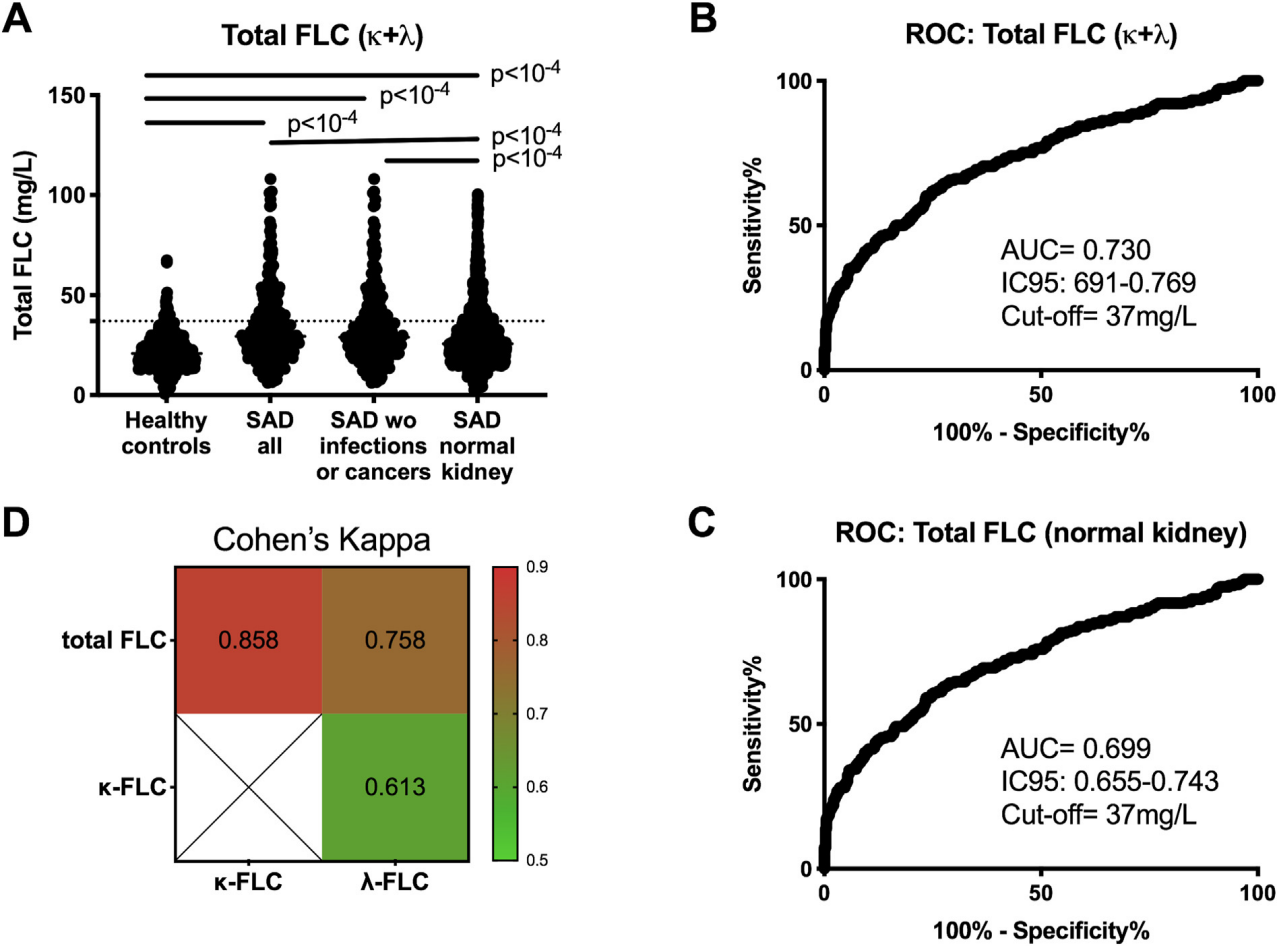
**Free light chain (FLC) levels are elevated in systemic autoimmune diseases (SAD). A.** Serum levels for total FLC (FLC-κ+λ) in controls (n ​= ​614), SAD patients (n ​= ​1979) and within the subgroups of SAD patients without infection and/or a history of cancer (n ​= ​1782), and with normal kidney function (n ​= ​1274). The dotted lines represent the selected cut-off fixed at 95% specificity: 37 ​mg/L for total FLC. **B:** ROC curves were performed in controls and SAD in order to determine the area under the curve (AUC) and cut-off values (95% specificity). **C:** ROC curves showing similar AUC characteristics when the SAD patient subgroup without renal impairment was selected instead of all SAD patients. **D:** Concordance assessment between total FLC (FLC-κ+λ), FLC kappa (FLC-κ), and FLC lambda (FLC-λ) using the Kappa coefficient of Cohen.

Total FLC (κ+λ) levels were evaluated by quantitative turbidimetry and, as illustrated in Fig. 1A, a significant elevated concentration was found in SAD as compared to HC (p < 10^−4^), while the FLC-κ/λ ratio did not differ from the HC ratio (Supplementary Fig. 1A). Next, when comparing total FLC between SAD and HC (Fig. 1B), the AUC of the ROC curves was 0.706, and the positivity fixed at 37 mg/L (sensitivity 29.3%) when using a 95% specificity cut-off, as recommended by the supplier. Of note (Fig. 1C and Supplementary Fig. 1B and C), total FLC was further selected instead of FLC-κ or FLC-λ based on AUC values of the ROC curves and the degree of agreement between total FLC and FLC-κ (Cohen's kappa = 0.858, IC95: 0.832–0.883) or FLC-λ (kappa: 0.759, IC95: 0.726–0.790) as such agreement was moderate between FLC-κ and FLC-λ (Cohen's Kappa = 0.613, IC95: 0.573 to 0.653). A correlation between total FLC and the IgG isotype (p = 2 × 10^−5^) was further observed, which was not the case with IgA or IgM (Supplementary Fig. 2). Total FLC levels were independent from sex, disease duration, and age at sample collection (Supplementary Fig. 3).

To complete such analysis and since FLC elevation in SAD can result from a polyclonal B-cell activation that may result from infection, cancers, and/or from a reduced clearance by impaired renal function, the SAD subpopulation without infection and cancer was evaluated (412 SLE, 338 SjS, 352 SSc, 349 RA, 85 APS, 92 MTCD and 145 UTCD) as well as the population with normal kidney function was individualized (294 SLE, 213 SjS, 276 SSc, 252 RA, 57 APS, 75 MTCD and 107 UTCD). In terms of AUC and cut-off values, the characteristics of the ROC assay was similar for total FLC when considering the SAD subpopulation with normal kidney function instead of all SAD individuals and SAD without infections and/or cancer (Fig. 1B and C and data not shown). Accordingly, we have concluded that FLC levels when detected over the elevated normal range in SAD resulted predominantly from an increase in polyclonal production independently from concurent infection and/or cancer and that reduced renal clearance, when present, amplifies this effect.

### 3.2 Total FLC prevalence in SAD and clinical associations

**Fig. 2:**
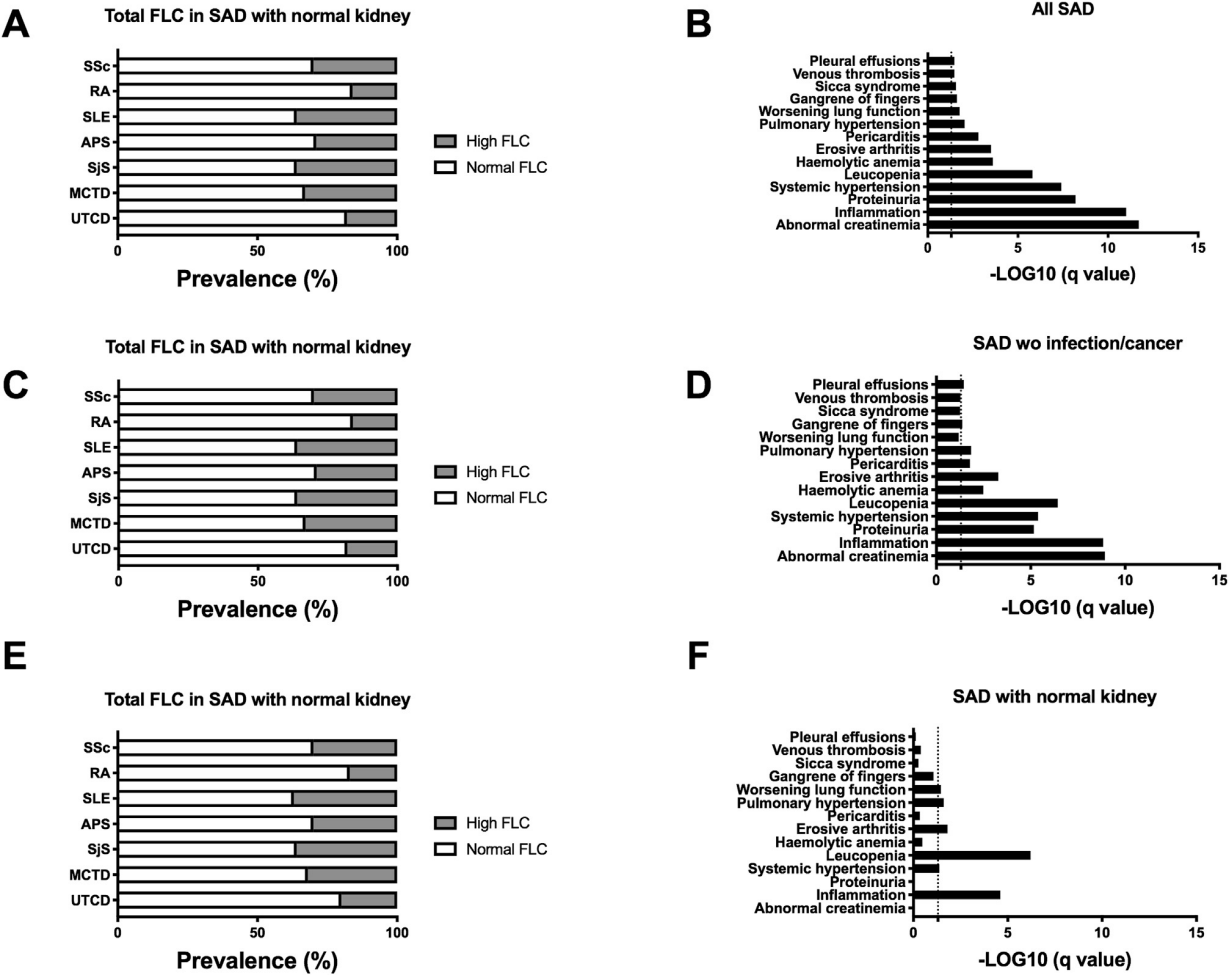
**Elevated free light chain (FLC) levels are reported in patients with systemic autoimmune disease (SAD) and associations with clinical manifestations**. **A:** Distribution of patients with elevated total FLC levels, results are expressed as percentages. **B:** Significant clinical parameters associated with elevated total FLC in all SAD patients are presented. **C/D**: Distribution and clinical associations in the subgroup of SAD patients without infection and/or cancer. **E/F**: Distribution and clinical associations in the subgroup of SAD patients with normal kidney function. Abbreviations: SLE: systemic lupus erythematosus; RA: Rheumatoid Arthritis; SjS: Sjögren Syndrome; SSc: Systemic Sclerosis; APS: Primary Antiphospholipid Syndrome; MCTD: Mixed connective tissue disease; and UTCD: Undetermined connective tissue disease. For statistical analysis a T-test comparing SAD with normal versus elevated FLC levels was used and adjusted false discovery rate values (q-values), with a threshold <0.05, were expressed as –log10 of the q value.

An excessive total FLC concentration (>37 mg/L) was determined in the 6 SAD subgroups ranging from 12% in RA, 14% in UCTD, 22% in SSc, 24% in SjS, 29% in MCTD and 30% in APS and SLE (Fig. 2A). Among the 54 clinico-biological parameters tested and the use of a post hoc FDR adjustment (Fig. 2B), the picture of SAD individuals with elevated total FLC levels was characterized significantly by abnormal creatinemia (p = 1.9 × 10^−19^), inflammation (p = 1.0 × 10^−11^), proteinuria (p = 5.7 × 10^−9^), hypertension (p = 4.0 × 10^−8^), leucopenia (p = 1.4 × 10^−6^), hemolytic anemia (p = 2.0 × 10^−4^), erosive arthritis (p = 3.0 × 10^−4^), pericarditis (p = 1.5 × 10^−3^), worsening lung function (p = 0.02), gangrene of the fingers (p = 0.02), *sicca* syndrome (p = 0.03), venous thrombosis (p = 0.03) and pleural effusion (p = 0.03).

However, and as one may argue that such associations resulted predominantly from infections, cancer and/or renal impairment, the analysis was repeated with the SAD subpopulations after exclusion of the individuals presenting infections/cancer and or presenting normal kidney function, which were determined to have an elevated total FLC level in the 6 SAD subgroups ranging respectively from 16 to 17% in RA, 18–20% in UCTD, 30-30% in SSc, 36-36% in SjS, 33-32% in MCTD, 29–30% in APS and 36–37% in SLE (Fig. 2C/E). While associations were similar when considering the whole SAD population and the subgroup without infection/cancer (Fig. 2D), or when regarding the subgroup with normal kidney function (Fig. 2F), major associations with inflammation (p = 2.7 × 10^−5^) and reduced leucopenia (p = 6.0 × 10^−5^), while moderate associations with systemic hypertension (p = 0.04), erosive arthritis (p = 0.02), pulmonary hypertension (p = 0.03) and worsening lung function (p = 0.04) remained. Altogether this supports the concept that elevated 10.13039/100006600FLC are commonly distributed among 10.13039/501100009237SAD patients regardless of kidney function, and this is predominantly related to the inflammatory status while independent from infection and cancer.

### 3.3 Correlation between total FLC, clinical disease activity and treatment

Regarding the association between FLC and disease activity, it appeared that patients with elevated FLC levels had a higher disease activity when considering SLE patients (SLEDAI: 4.2 ± 0.6 in normal FLC SLE *versus* 6.7 ± 0.8 in high FLC SLE, p = 0.01), SjS patients (ESSDAI: 3.6 ± 0.4 in normal FLC SjS *versus* 5.7 ± 0.7 in high FLC SjS, p = 0.01), and such associations were not observed when using a global VAS approach.

As a worsening lung function was statistically associated with elevated FLC levels, the lung capacity was explored further in SSc patients, which revealed a median loss of FVC among SSc patients with elevated FLC levels (97.2 ± 1.8% in normal FLC SSc *versus* 84.3 ± 2.9% in high FLC SSc, p = 0.0002). No differences were observed with regards to DLCO and the ratio FLC/DLCO, two factors known to occur in a more advanced stage of fibrosis and in response to a strong inflammatory process.

**Fig. 3:**
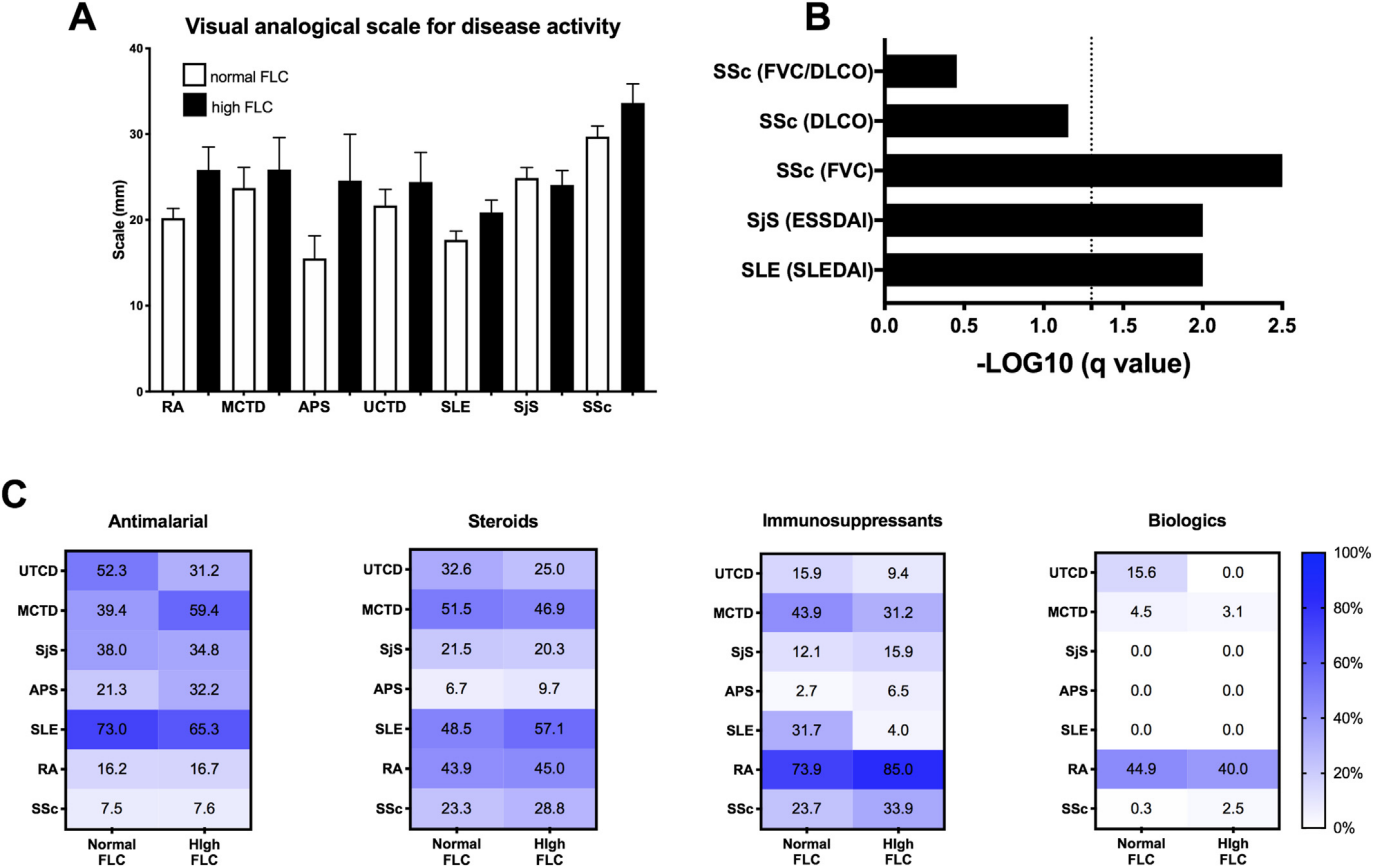
**Free light chain (FLC) levels are associated with increased disease activity in patients with systemic autoimmune diseases (SAD), pulmonary functions in systemic sclerosis (SSc) and independent of current treatments. A.** Visual analogical scale representing the disease activity scoring system designed for the PRECISESADS study, according to the FLC status of SLE, RA, SjS, SSc, APS, MTCD and UTCD patients. **B.** Pulmonary function using FVC, FLCO and FVC/FLCO ratio was assessed for SSc; and the disease activity scores ESSDAI and SLEDAI, were calculated and only available for SjS and SLE patients, respectively. For statistical analysis, a T-test comparing SADs with normal FLC versus elevated FLC levels was used and adjusted false discovery rate values (q-values) with a threshold <0.05 were expressed as –log10 of the q value. **C.** Heat-map displaying treatments (anti-malarial, steroids, immunosuppressant and biological) taken by SADs patients according to their serum FLC levels. Results are expressed as percentages of patients currently taking the corresponding treatment. Abbreviations: FVC: forced vital capacity; DLCO: Diffusing Capacity for Carbon Monoxide; ESSDAI: EULAR Sjogren's Syndrome Disease Activity Index; SLEDAI: systemic lupus erythematosus disease activity index; SLE: systemic lupus erythematosus; RA: Rheumatoid Arthritis; SjS: Sjögren's syndrome.

The dichotomy between normal and high FLC subgroups in SAD was also studied in relation to the treatment (Fig. 3C). No statistical differences for the 6 SAD studied were reported when considering the different therapeutic approaches used in SAD: antimalarial, steroids, immunosuppressant and biologics.

### 3.4 Total FLC concentrations in relation to autoantibodies and complement in SAD

**Fig. 4:**
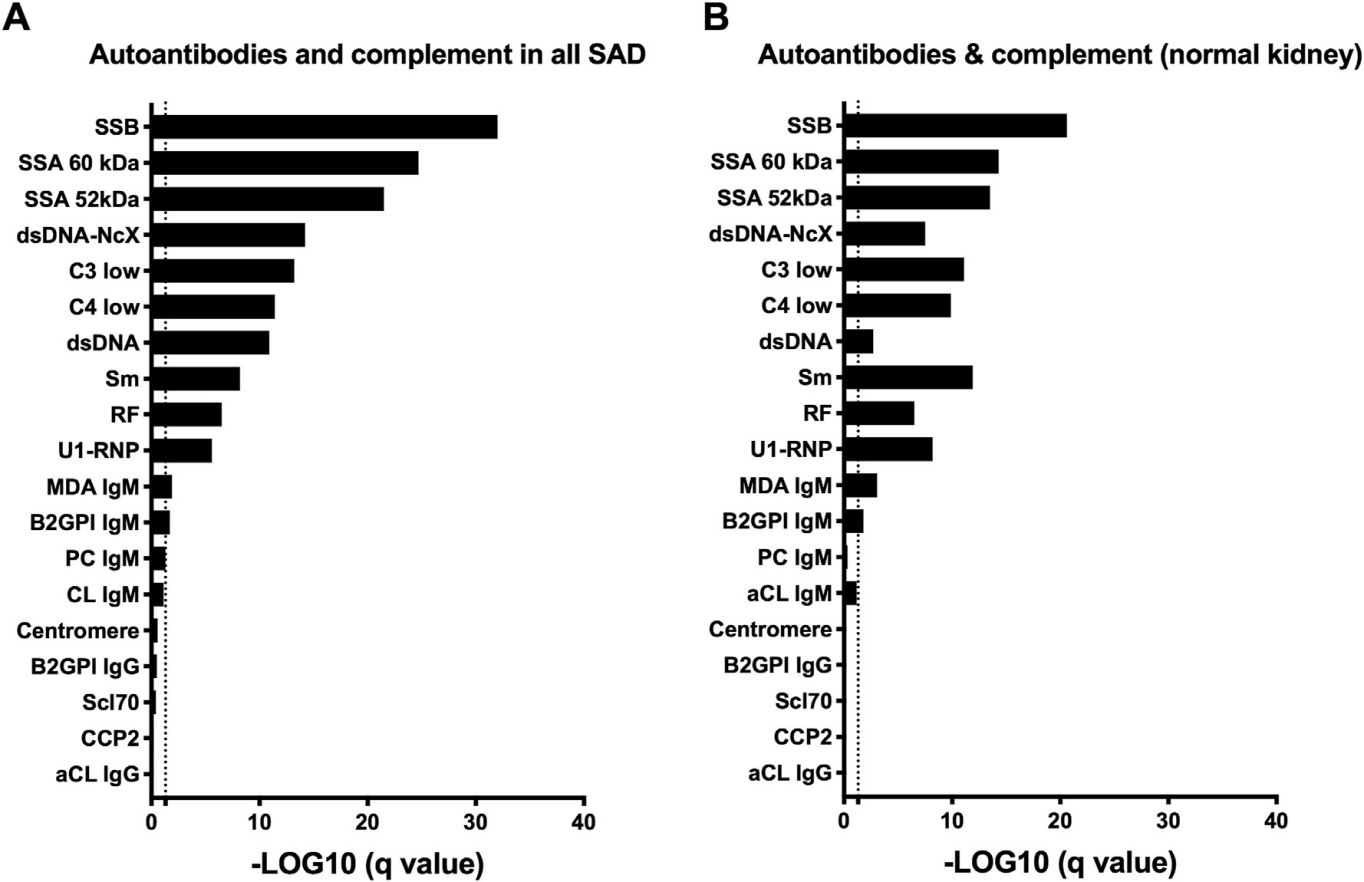
**Free light chain (FLC) levels are correlated with a subset of autoantibodies and complement reduction in systemic autoimmune diseases (SAD)**. **A:** all systemic autoimmune disease (SAD) patients. **B:** subgroup of SAD patients with normal renal function. Abbreviations: SSA/B: anti-sicca syndrome A or B autoantibodies (Ab); dsDNA-NcX: anti-DNA/nucleosome or anti-chromatin Ab; C3/C4 low: complement faction C3c/C4 with a negative correlation (low); dsDNA: anti-double stranded DNA Ab; Sm: anti-Smith Ab; RF: rheumatoid factors; U1-RNP: anti-U1 ribonucleoprotein Ab; MDA: anti-malondialdehyde modified LDL Ab; B2GPI: beta-2 glycoprotein I Ab; PC: anti-phosphatidyl choline Ab; aCL: anti-cardiolipin Ab; Centromere: anti-centromere Ab; Scl70: anti-Scl70 Ab; CCP2: anti-citrullinated peptide generation 2 Ab; IgG: immunoglobulin G; IgM: immunoglobulin M. For statistical analysis pairwise Pearson's correlations between total FLC and biological parameters were calculated and the adjusted false discovery rate values (q-values) expressed using the –log10 of the q value.

In order to determine whether FLC levels were associated with a large panel of SAD-associated autoantibodies and complement fractions C3/C4 (Fig. 4A), a correlation analysis was performed and followed by post hoc FDR adjustment. Accordingly, 10/17 (58.8%) autoantibodies tested were positively correlated with FLC levels while a negative correlation was reported for C3 and C4 levels (p < 6.1 × 10^−14^ and 4.0 × 10^−12^, respectively). Interestingly, two groups of autoantibodies can be distinguished according to the correlation with FLC: one correlated with FLC that includes anti-RNA binding proteins (SSB, SSA 52/60 kDa, Sm, U1-RNP; 1.9 × 10^−40^<p < 2.2 × 10^−6^), anti-dsDNA associated or not with histones to form chromatin (p = 6.5 × 10^−15^ and 1.1 × 10^−11^) plus RF (p = 3.3 × 10^−7^) and another group not or modestly correlated with FLC such as RA-associated (CCP2, p = 0.608), SSc-associated (Scl70, p = 0.386; centromere, p = 0.272), APS-associated (aCL/β2-GPI IgG/M; 0.02 < p < 0.708), and thrombosis-associated (MDA IgM, p = 0.01; PC IgM, p = 0.05) autoantibodies. Since some of these autoantibodies were more prevalent in SAD patients with kidney involvement (e.g. dsDNA, Sm), the same analysis was repeated in SAD individuals without infection and cancer (data not shown) and in SAD with normal kidney function (Fig. 4B) confirming the correlation between FLC levels with anti-RNA binding protein autoantibodies, anti-dsDNA autoantibodies, RF and a reduction of the complement fractions C3 and C4.

### 3.5 Total FLC in relation to the IFN signature

**Fig. 5:**
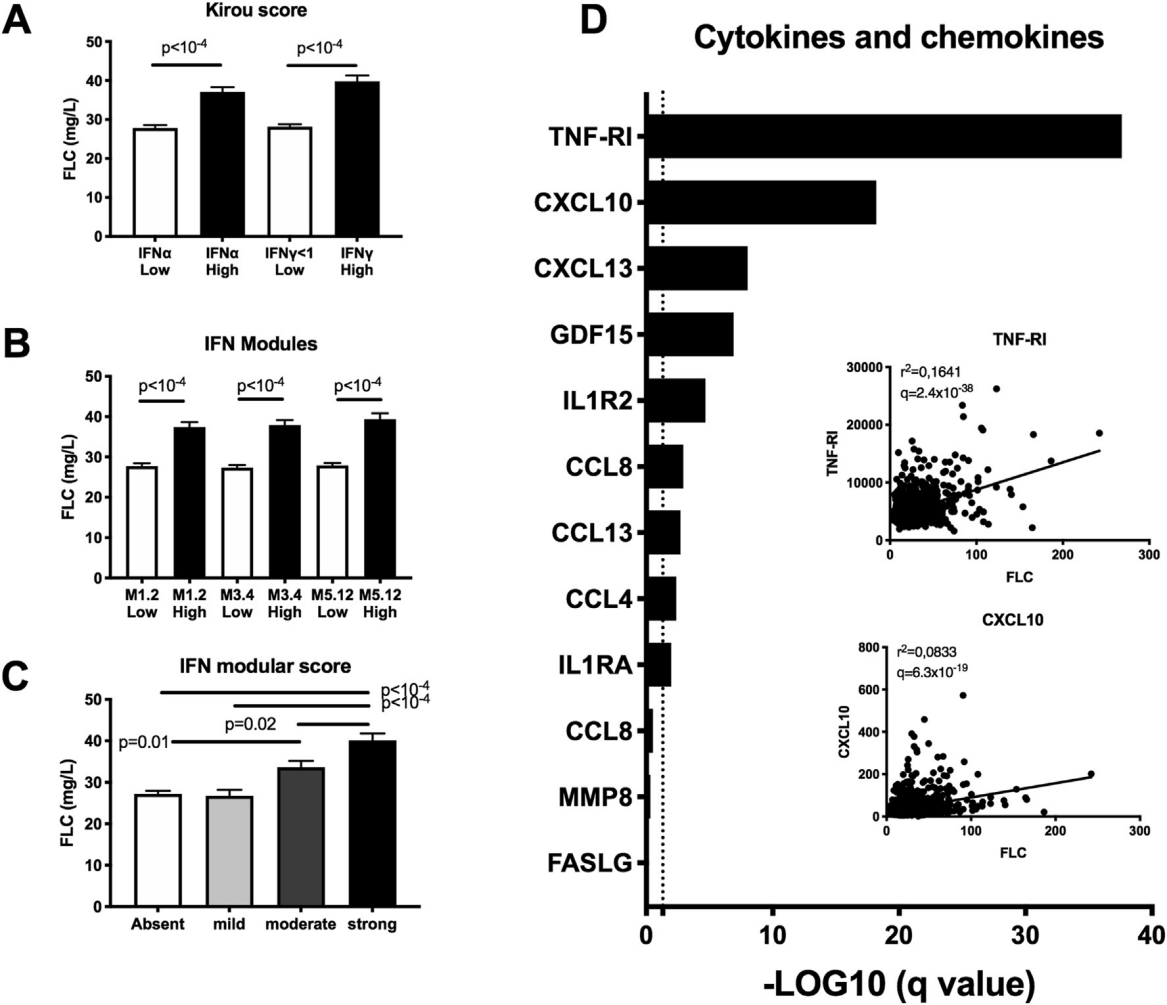
**Elevated levels of free light chains (FLC) found in patients with systemic autoimmune disease (SAD) are associated with an interferon (IFN) signature and pro-inflammatory cytokines and chemokines**. Serum total FLC levels in patients with SAD according to their IFN signature based on (**A**) Kirou's score that evaluates IFNα and IFNγ signatures; (**B**) modular score based on 3 co-clustered gene sets: M1.2, M3.4 and M5.12. The M1.2 was induced by IFNα/β, while both M1.2 and M3.4 transcripts were upregulated by IFNα/β and IFNγ; (**C**) the number of positive modules: 0 (absent); 1 (mild); 2 (moderate); and 3 (strong). High Kirou's scores and IFN modules score were determined based on the mean value ​+ ​2 standard deviations from the control population. **D:** Free light chain levels are correlated with inflammatory cytokines and chemokines in patients with SAD. For statistical analysis pairwise Pearson's correlations between total FLC and pro-inflammatory cytokines and chemokines were calculated and the adjusted false discovery rate values (q-values) expressed using the –log10 of the q value. Correlation curves with Spearman's rho and q values are also displayed for TNF-R1 and CXCL10.

The presence of autoantibodies targeting dsDNA and RNA binding proteins (e.g. SSA, SSB, Sm epitopes) in several cohorts of SAD is associated with the presence of an IFN signature. Accordingly (Fig. 5), we next compared FLC levels in 1271 SAD patients to the IFN scores and modules established by Kirou and Obermoser, respectively [23,24]. First, using the IFNα and IFNγ Kirou score, SAD patients with a negative IFNα/γ score had lower FLC levels (p < 10^−4^), which is consistent with the possible implication of both type I and type II IFNs in FLC production. Such a prediction was further supported by the elevated level of 10.13039/100006600FLC observed in the type I activity module M1.2 and in the type I/II activity modules M3.4 and M5.12. In addition, the number of IFN modules is important since FLC levels were significantly higher in the moderate module (p = 0.01) and even more in the strong module (p < 10^−4^). For 866 SAD patients, the serum level of 12 cytokine/chemokine was determined showing a significant correlation for 9/12 (75%), such as TNF-RI (p = 2.5 × 10^−38^) and CXCL10 (IP-10, p = 6.4 × 10^−19^), which belong to the IFN type I and II, except for CCL18 (type II, exclusively), according to the Interferome V2.01 database (http://www.interferome.org).

## 4 Discussion

Systemic autoimmune diseases share common pathophysiological features such as chronic inflammation and an IFN signature leading to the initiation of Th1-type immunity, B cell hyperactivation and differentiation into autoantibody-secreting plasma cells [26]. The consequent polyclonal production is associated with excessive secondary production of FLC, which has already been described in several SADs such as SLE, RA, SSc, or SjS [[27], [28], [29], [30], [31]]. Here, we report the results of a large multi-center cross-sectional study involving 1979 patients and 614 HC establishing the relationship between FLC and several parameters such as clinical manifestations, disease activity, treatment influence, autoantibody production, and interferon-related inflammation.

Because of their rapid turnover, a high level of FLC has been proposed as useful real-time marker for assessing disease activity and B cell hyperactivity, in contrast to total immunoglobulins, which possess a half-life of approximately 3 weeks depending on the isotype [32]. Such assertion needs however to satisfy several criteria, first to assess a polyclonal production of immunoglobulins the ratio κ/λ needs to be similar between patients and HC, which was observed in our study explaining that monoclonality was not further looking for. Second, elevated FLC levels in response to infection and cancer were not retrieved in our study suggesting a minor impact of these two co-morbidity factors in SAD on FLC levels. Third, glomerular function is also critical as a defective clearance is able to increase FLC levels. However, such effect is suspected to occur at a level higher than the elevated cut-off fixed for FLC-κ, FLC-λ, and total FLC (κ+λ) based on the comparative analysis performed between total SAD patients and SAD patients with normal kidney function. Accordingly and as previously reported [7], an excess of total FLC was retrieved in all SAD patient groups, ranging from 12 to 17% in RA, 14–20% in UCTD, 22–30% in SSc, 24–36% in SjS, 29–32% in MCTD, 30% in APS, and 30–37% in SLE when considering total SAD patients and SAD patients with normal kidney function, respectively.

Elevated FLC levels were also associated with the occurrence of clinical and biological manifestations and, for such analysis, it's important to consider one group of associations independent from renal impairment that implies inflammation such as pulmonary injury and erosive arthritis; and one group associated with renal impairment such as hypertension, pericarditis, and venous thrombosis that are particularly relevant in SADs. Altogether, elevated FLC levels are associated with the occurrence of clinical manifestations associated with a chronic inflammatory process, in accordance with previous data obtained in SLE, SSc and SjS cohorts [27,29,30]. This is consistent with the observed association reported between FLC levels and an alteration of the pulmonary capacity (FVC) due to inflammation in SSc. The level of FLCs could then be useful in predicting the occurrence of pulmonary symptoms and lung fibrosis, which is the primary cause of mortality in SSc patients along with cardiac manifestations [33]. In a previous report, high FLC levels in SSc were also associated with lung involvement and with a higher degree of inflammation [28]. As a consequence, it's not surprising that we have observed in SLE and SjS patients a significant association between elevated FLC levels and higher disease activity as assessed by SLEDAI and ESSDAI scores, respectively. Search for an association between FLC levels and disease activity was not performed in the other disease groups as such information was facultative at inclusion and then available only for SLE and SjS.

Subsequently, FLCs have also been associated with specific autoantibodies, including anti-RNA binding protein antibodies (anti-SSB, anti-SSA, anti-Sm, and anti-RNP), as well as anti-DNA-NcX, anti-dsDNA, and RF. In contrast, the other autoantibodies tested (anti-CCP, anti-β2GPI, anti-PC, anti-CL, anti-centromere, and anti-Scl70) demonstrated poor or no association with increased FLC and the same results were obtained in patients with normal kidney function. One explanation for such a dichotomy is related to the capacity of the FLC-associated autoantibody group to form immune complexes (e.g. with apoptotic cells), which is known to activate the complement pathway and to increase monocyte IFN type-I production that promotes, in turn, plasmablast differentiation and immunoglobulin production [34,35]. Such model observed in individuals infected with the hepatitis C viruses [36], needs further exploration in SAD.

In addition, an important negative correlation between FLC and complement fractions was noted. An effect of FLC to directly promote complement consumption has been proposed previously, involving the binding of FLC to factor H, which accelerates the decay of the alternative complement compound C3 convertase [10]. This mechanism could be of particular interest in SADs in which complement activation is associated with severe clinical manifestations. In SLE, the activation of both the classical and alternative complement pathways induces the deposition of various complement components together with immunoglobulins and is responsible for lupus glomerulonephritis, thus indicating an active disease [37]. In addition, complement activation is described in APS and can be responsible for fetal loss or fetal growth retardation. In RA, there is also an accelerated consumption and a hyperproduction of complement fractions described in synovial fluid. Finally, hypocomplementemia is frequently described in SjS and is associated with poor outcomes [38].

Finally, a strong link between FLC and inflammation has been demonstrated through the analysis of the IFN signature and selected proinflammatory cytokines. Two IFN scores were applied [23,24] and both revealed a correlation between FLC and increased production of IFN type 1 and type 2 molecules. Higher interferon activity has already been described in several SADs [2]. In SLE, activation of the IFNα pathway identifies a subgroup of SLE patients with distinct serologic features and a higher disease activity. It is interesting to note that the implicated autoantibodies are the same as reported in our study and are autoantibodies directed against RNA-binding proteins [23,39], suggesting the interplay between IFN driven inflammation and autoantibody production as well as FLC production in an amplification loop to reflect a clinically-active disease. IFNβ and IFNγ signatures are also reported in SLE patients and have led to the stratification of patients according to their IFN signature. Patients having absent to low IFN score (0–1 modules) i.e. IFNα-only signature can be opposed to patients with moderate to high modular IFN scores (2–3 modules) i.e. IFNα, -β and -γ signatures. In the latter case, a specific association was made with active renal disease, anti-dsDNA presence and hypocomplementemia that is also noted in patients with elevated FLC in our study [25,40]. Indeed, when looking at our results, we obtained the highest link with FLC when all the modules were significantly elevated. In RA, a high level of 10.13039/501100007072IFN type I predicted the response to TNFα blockade, but not to rituximab, a B cell depleting monoclonal antibody, supporting the fact that 10.13039/501100007072IFN type I signature in RA induces a high inflammatory activity, but not an elevated autoantibody production by B cells based on the independence observed between 10.13039/100006600FLC levels and anti-10.13039/100005550CCP production [41]. Finally, IFNγ was also pathologically increased in UTCD and MTCD [42,43].

To further support this link between 10.13039/501100007072IFN, inflammation and 10.13039/100006600FLC synthesis, several inflammatory cytokines belonging to the type 1 and type 2 10.13039/501100007072IFN families were measured and were significantly correlated with higher total 10.13039/100006600FLC levels. Among them, the two most important cytokines retrieved by our study were TNF-RI and CXCL10, both known to be overexpressed in SLE B cells, but such analysis was not exhaustive and needs further exploration.

The key role played by TNFα in RA was studied extensively through therapeutic use of anti-TNFα monoclonal antibodies like infliximab or the use of fusion proteins trapping soluble TNFα, like etanercept. TNFα can bind to two cell-surface receptors, TNF-RI and TNF-RII. The presence of these receptors in sera of RA patients was shown to be higher as compared to controls and was associated with a higher disease activity score (DAS28) and an abnormal inflammation status [44]. In an *ex-vivo* cell culture model of synovial membrane tissue obtained from RA patients, a dual activity between TNF-RI and TNF-RII was observed together with a predominantly pro-inflammatory role played by TNF-RI in RA [45]. TNF-RI was also described as increased in B cells from SLE patients as compared to controls [46], and was of particular interest to predict disease flares [47]. Of note, the use of a TNF-RI inhibitor in a mouse model of lupus was effective in preventing skin lesions [48]. Less evidence has been found for SjS, but TNF-RI is still detected at significantly higher rates in patients than in controls [49]. Last but not least, TNF-RI was shown to be up-regulated in dermal T cells from patients with diffuse cutaneous SSc. Those lymphocytes were more prone to generate a proinflammatory microenvironment after CD3/CD28 T cell engagement and in the presence of a co-stimulation with TNF-RI ligands. The microenvironment triggered by dermal T lymphocytes induced high and sustained type I collagen expression, supporting a role for the TNF-RI pathway in induction of fibrosis [50]. This is in agreement with our results where an altered pulmonary function in those SAD patients with elevated total FLC levels, is possibly due to the associated inflammation and TNF-RI pathway.

On the other hand, CXCL10 is a cytokine responsible for the recruitment and activation of Th1 effectors, predominantly induced by IFNγ. CXCL10, in particular, is involved in the maintenance of chronic pathological inflammation, and abnormal secretion of this cytokine has been described in several SADs. In RA, the expression of CXCL10 was demonstrated to be elevated in the serum and joints of patients [51]. In a murine model, it was shown that CXCL10 participates in bone destruction by allowing up-regulation of RANKL on CD4 T cells that, in turn, increases CXCL10 production by osteoclast progenitors, thus establishing a positive feedback loop between the two cell types. CXCL10 induces the activation and differentiation of osteoclasts in a dose-dependent manner. The use of an anti-CXCL10 antibody blocks osteoclast differentiation, thereby preventing the progression of arthritis in mice that present with fewer histological sequelae than controls [52]. In SLE, CXCL10, overexpressed in SLE B cells, was also associated with more severe forms, anti-dsDNA positivity and lowered complement, which is a profile that also emerges from our data [53]. In a murine model of autoimmune sialadenitis mimicking SjS, the use of an antagonist of CXCL10 prevents the progression of the disease with a decrease in IFNγ production, underlining the link between the two cytokines [54]. Regarding SSc, an analysis of patients showed that besides Th2 chemokines (CCL17 and CCL22), CXCL10 was detected in significantly higher proportions in patients with diffuse or localized SSc than in normal controls [55]. Finally, a clear role for CXCL10 produced by macrophages to induce plasma cells differentiation and production of antigen-specific antibodies *in vivo* has recently been demonstrated, highlighting a mechanism for the secondary production of FLC through an inflammatory cytokine related to the IFN signature [28]. Of note, CXCL10 binds to CXCR3 on memory B cells to terminate their differentiation into plasma cells, which is significantly increased by IFNγ [56], making a full link between IFN-mediated inflammation and B cell differentiation.

Those results are consistent with the participation of IFN type I and type II as well as several proinflammatory cytokines to induce more severe diseases through FLC synthesis. Elevated FLC would then accurately mirror both inflammation as well as pathological B cell activation in SAD patients.

## 5 Conclusion

This is the first study to determine the role of FLC on such a large scale of SAD to our knowledge. The major findings are related to the facts that (i) immune stimulation rather than impaired kidney function, infection or cancer increases polyclonal FLC in SAD; (ii) Elevated FLC levels are associated with disease activity, inflammation, and a specific profile of autoantibodies; and (iii) a relationship between elevated FLC levels and a strong IFN signature was further revealed. FLC then account for adaptive immunity by reflecting autoantibody production and B cell activity, but also for innate immunity through its induction by IFN signaling. FLC measurement, especially in patients with stable renal function, would then be interesting to evaluate disease activity in real time with an easy and reliable assay. It would also be interesting to complement these observations with a longitudinal study to evaluate the variation of FLCs over time according to disease activity and inflammatory parameters, and thus define the value of FLC in predicting relapses and/or therapeutic response.

## Authorship contribution statement

EB, PLM, MOB, MAR, YR: Formal analysis and writing original draft; EB, CLG, MM, MOB, GB, ZM, SB, RL: Investigation; MAR, JF: Project administration; and MAR: Coordination of the whole project and the person behind PRECISESADS ideas.

## Declaration of competing interest

All authors declare that they have no conflict of interest.
