# Supplementary material for: An elevated polyclonal free light chain level reflects a strong interferon signature in patients with systemic autoimmune diseases
Source: J Transl Autoimmun. 2021 Mar 2;4:100090. doi: 10.1016/j.jtauto.2021.100090 (PMC8010703; doi:10.1016/j.jtauto.2021.100090)
Supplement: Multimedia component 2 [file mmc2.docx]

**CROSS SECTIONAL COHORT CRF DESCRIPTION**

**Demographic and diagnosis**

- Patient ID

- Date of birth

- Date of Consent (Yes/No → Date)

- Gender (Scroll Down)

-M/F

-Ethnic origin (standard form)

- Current diagnosis based on available criteria (Scroll Down)

-Rheumatoid arthritis (RA)

-Systemic Lupus Erythematosus (SLE)

-Systemic Sclerosis (SSc)

-Sjogren Syndrome (SjS)

-Mixed Connective Tissue Disease (MCTD)

-Primary Antiphospholipid Syndrome (PAPs)

-Undifferentiated Connective Tissue Disease (UCTD)

-Disease onset (Date)

*Diagnostic criteria used in the inclusion of patients will be included in a note*

-RA: 2010 ACR/EULAR classification criteria (*Ann Rheum Dis* 2010;69:1580-1588)

-SLE: 1997 update of 1982 ACR criteria (*Arthritis Rheum* 1997;40:1725).

-SSc: ACR/EULAR 2013 classification criteria (*Ann Rheum Dis*. 2013;72:1747-55)

-SjS: AECG SjS classification criteria (*Ann Rheum Dis* 2002;61:554–8)

-MCTD: Alarcon-Segovia criteria for MCTD (*J Rheumatol* 1989;16:328-334)

-PAPs: International consensus statement on an update of the classification criteria for definite PAPs (J Thromb Haemost, 2006;4: 295–306)

-UCTD: patients with clinical features of SADs not fulfilling any of the above or any other SADs criteria for at least 2 years + Presence of ANA ≥ 1:160 with or without specific autoantibodies.

Patients fulfilling 3 out of 4 SLE classification criteria and patients with Early Systemic Sclerosis (*J Rheumatol.* 2001, 28:1573-6*) should not* be classified as UCTD

The next paragraph will be adapted once agreed in the detailed section

***General symptoms and inflammation***

-Fever (Yes/No)

-Abnormal inflammatory indexes No/Past/Present/unknown

-Hypergammabulinemia No/Past/Present/unknown

-Disease activity (Entry box)

***General laboratory***

-Abnormal CBC, including:

-Haemolytic anemia (Past/Present/No/Unk)

-Reduced White Blood Cells count (Past/Present/No/Unk)

*­*-Reduced platelet count (Past/Present/No/NAUnk)

-Reduced C3 levels (Past/Present/No/Unk)

-Reduced C4 levels (Past/Present/No/Unk)

***Vascular***

-Ischemic digital ulcers/Pitting scars (Past/Present/No/Unk)

-Gangrene of the fingers (Past/Present/No/Unk)

-History of Raynaud’s phenomenon (Yes/No/Unk)

-Arterial/Venous thrombosis (Past/Present/No/Unk)

-History of recurrent miscarriage or pregnancy complications (Yes/No/NA)

***Gastrointestinal***

-History of esophageal Reflux disease (Yes/No/Unk)

-Oesophageal dismotility (Yes/No/Unk)

-Abnormal liver function tests (Past/Present/No/Unk)

***Skin and mucosa***

-Cutaneous Lupus, active (Past/Present/No/Unk)

-Cutaneous Lupus, chronic (Past/Present/No/Unk)

-Apthous ulcers (Past/Present/No/Unk)

-Photosensitivity (Past/Present/No/Unk)

-Calcinosis cutis (Yes/No/Unk)

-Skin fibrosis (Past/Present/No/Unk)

-Puffy Fingers (Past/Present/No/Unk)

-Telangectasia (Yes/No/Unk)

-Subcutaneous nodules (Past/Present/No/Unk)

-Sicca syndrome (Yes/No/Unk)

***Heart***

-Pericarditis (Past/Present/No/NA)

-Pulmonary hypertension on Echo (Past/present/No/Unk

)

-Confirmation of pulmonary arterial hypertension by Cath (Yes/No/Unk)

-Hypertension (Yes/No/Unk)

-Arrythmias (Past/Present/No/Unk)

-Valvle lesions (Yes/No/Unk)

***Lung***

-Basilar crackles (Yes/No/Unk)

-Pleural effusions (Past/Present/No/Unk)

-Pulmonary fibrosis (Yes/No/Unk)

-Functional ventilatory restriction (Yes/No/Unk)
-Worsening lung function (Yes/No/Unk)

***Muscle-skeletal***

-Arthritis (Past/Present/No/Unk)

-Erosive arthritis (Yes/No/Unk)

-Muscle weakness (Past/Present/No/Unk)

-Evidence of inflammatory myopathy (Yes/No/Unk)

***Kidney***

-Abnormal Creatininine (Past/Present/No/Unk)

-Abnormal urine analysis (Past/Present/No/Unk)

-Urine proteins (Past/Present/No/Unk)

-Proteinuria (No/Moderate/Severe/Unk)

-Biopsy proven nephritis (Yes/No/Unk)

-Abnormal lipid profile (Past/Present/No/Unk)

***Nervous system***

-Involvement of the CNS involvement (Past/Present/No/unk)

-Involvement of the PNS (Past/Present/No/unk)

***Therapy***

-Antimalarials (Yes/No)

-Immunosoppressants (yes/No)

Systemic antibiotics (yes/No)

-Biologicals (Scroll Down)

-No

-Anti-TNF (any molecule)

-Tocilizumab (or other anti-IL6)

-Abatacept

-Steroids, dose (Yes/No → Dose)

**Comorbidities (Yes/No/unk)Definitions of the CRF items**

| **General** | |
| --- | --- |
| **Field** | **Definition** |
| Yes/No/NA | The Yes/No fields refers to the actual situations, that is findings that have been observed during the recent past unless where otherwise specified.  If the assessment of the item has not been done in the specified time-frame, thick the “NA” option.  Case-specific definitions are provided for each item. In summary:  -Abnormal inflammatory indexes: Yes = within 4 weeks  -Hypergammaglobulinemia: Yes = within 12 weeks (3 months)  -Pumonary hypertension on Echo: Yes = within 6 months  -Pulmonary fibrosis, ground glass opacities: YES = within 12 months  -Functional ventilatory restriction: Yes = within 6 months  -Worsening lung function: Yes = within 12 months  For non-receding conditions no temporal limit will be applied and “Yes” would mean “Ever” and “No” would mean “Never”.  Case-specific definitions are provided for each item. In summary:  -Raynaud’s Phenomenon: Yes = Ever  -Recurrent miscarriage or pregnancy complication: Yes = Ever  -Esophageal reflux disease: Yes = Ever  -Oesophageal dismotility: Yes = Ever  -Calcinosis cutis: Yes = Ever  -Telangectasia: Yes = Ever  -Sicca Syndrome: Yes = Ever  -Pulmonary arterial hypertension by cath: Yes = Ever  -Hypertensions: Yes = Ever  -Valve lesions: Yes = Ever  -Pulmonary fibrosis, honeycombing: Yes = Ever  -Erosive arthritis: Yes = Ever  -Evidence of inflammatory myopathy: Yes = Ever  -Biopsy proven nephritis: Yes = Ever |
| Past/Present/No/NA | The term “past” refers to a condition that was observed *before the prespecified time* from the evaluation and that is no longer present orwhich has not been verified since the observation.  The term “present” refers to a condition that is still observable. |

| **Demographics** | | |
| --- | --- | --- |
| **Field** | **Definition** | **Options** |
| Disease onset | Approximate date at the onset of the first symptom clearly attributable to the disease; for systemic sclerosis the first non-Raynaud symptoms. | Date |

| **General symptoms and inflammation** | | |
| --- | --- | --- |
| **Field** | **Definition** | **Options** |
| Fever | Axillary (armpit) temperature ≥ 38° for at least one week not due to concurrent infections. | Yes/No/unk  Unk = not verified |
| Abnormal inflammatory indexes | Presence of any of the following within *4 weeks*; concurrent infections should be ruled out.  ESR > 30 mm/h  and/or  CRP > ULN | No/past/present/unknown  Past : observed and resolved or not verified since an observation of more than 4 weeks  Present : within 4 weeks and not resolved  No : never observed  Unknown : never evaluated |
| Hypergammaglobulinemia | Presence of any of the following within *12 weeks*  Serum IgG > ULN  and/or  Gammaglobulin > 20% | No/past/present/unknown  Past : observed and resolved or not verified since an observation of more than 12 weeks  Present : within 12 weeks and not resolved  No : never observed  Unknown : never evaluated |
| Disease Activity | 0-100 Visual analogue scale based on the physician’s judgement | Entry box  At the visit |

| **General Laboratory** | | |
| --- | --- | --- |
| **Field** | **Definition** | **Options** |
| Hemolytic anemia | Hb<13.7 g/dL for Males aged 20-60 yrs or Hb<13.2 for males aged ≥ 60 yrs or Hb<12.2 g/dL for Females  and  Positive direct Coombs' test  and  Serum haptoglobin < LLN | Past/Present/No/Unk  Past : observed and resolved or not verified since an observation of more than 4 weeks  Present : within 4 weeks and not resolved  No : never observed  Unknown : never evaluated |
| Reduced White Blood Cell Count | WBC ≤ 3000 cells/mm^3^  and/or  Lymphocytes < 1000/mm^3^  and/or  Neutrophils < 1500/mm^3^ | Past/Present/No/Unk  Past : observed and resolved or not verified since an observation of more than 4 weeks  Present : within 4 weeks and not resolved  No : never observed Unknown : never evaluated |
| Reduced Platelet count | PLT ≤ 100,000/mm^3^ | Past/Present/No/Unk  Past : shown more than 4 weeks ago and resolved or not verified since an observation of more than 4 weeks  Present : within 4 weeks and not resolved  No : never observed  Unknown : never evaluated |
| Reduced C3 | C3 levels < LLN | Past/Present/No/Unk  Past : observed and resolved or not verified since an observation of more than 4 weeks  Present : within 4 weeks and not resolved  No : never observed  Unknown : never evaluated |
| Reduced C4 | C4 levels < LLN | Past/Present/No/Unk  Past : observed and resolved or not verified since an observation of more than 4 weeks  Present : within 4 weeks and not resolved  No : never observed  Unknown : never evaluated |

| **Vascular** | | |
| --- | --- | --- |
| **Field** | **Definition** | **Options** |
| Ischemic digital ulcers or pitting scars | Ischemic demarcation with loss of epithelial substance extending to the dermis, not thought to be due to trauma.  Digital pitting scars are depressed areas at the tips with hyperkeratosis as a result of ischemia, rather than trauma or exogenous causes. | Past/Present/No/Unk  Past = observed and resolved  Present = at the visit  No = not observed  Unk: not verified |
| Gangrene of the fingers | Death and decay of the fingers | Past/Present/No/Unk Past = observed and resolved  Present = at the visit  No = not observed  Unk: not verified |
| History of Raynaud's Phenomenon | History of self report or reported by a physician with at least a two-phase color change in finger(s) or toe(s) consisting of pallor, cyanosis and/or reactive hyperemia in response to cold exposure or emotion; usually one of these phase is pallor. | Yes/No/Unk  Non receding condition  Yes=ever  No = never  Unk = not verified |
| Arterial/venous thrombosis | Any event, in any anatomical district (documented by imaging or histology) | Past/Present/No/Unk  Past : observed and resolved or not verified since observation of more than 4 weeks  Present : within 4 weeks and not resolved  No : never found  Unknown : never evaluated |
| Recurrent miscarriage or pregnancy complication | Any of the following conditions in the medical history.  Loss of ≥ 3 pregnancies before the 10th week of gestation after exclusion of chromosomal abnormalities or maternal anatomic abnormalities  or  At least 1 unexplained death of a morphologically normal fetus at or after the 10th week of gestation.  or  At least 1 premature birth of a morphologically normal neonate before the 34th week of gestation due to eclampsia or severe pre-eclampsia. | Yes/No/Unknown/NA  Yes = ever  No = never  Unknown := not verified  NA refers to “men” |

| **Gastrointestinal** | | |
| --- | --- | --- |
| **Field** | **Definition** | **Options** |
| History of esophageal reflux disease | History of persistent (> 2 months) symptoms of reflux disease attenuated by the use of PPI of anti-H_2_ antagonists. | Yes/No/Unk  Non receding condition  Yes = ever  No= never  Unk : not verified |
| Oesophageal dismotility | Reduced-amplitude or absent peristaltic contractions in the distal third of the esophagus and normal or decreased lower esophageal sphincter pressure as assessed by manomentry  and/or  Dilatation and loss of peristaltic contractions in lower esophagus as assessed by Barium swallow x-ray. | Yes/No/Unk  Non receding condition  Yes :=confirmed by manometry or Barium swallow X ray at any time  No if not shown by the exam performed  Unk : manometry or Barium swallow X ray never performed, irrespective of clinical signs/symptoms |
| Abnormal liver function tests | AST ≥ 3 times ULN  and/or  ALT ≥ 3 times ULN  and/or  gGT ≥ 3 times ULN  and/or  ALP ≥ 3 times ULN | Past/Present/No/Unk  Past : observed and resolved or not verified since an observation of more than 4 weeks  Present : within 4 weeks and not resolved  No : never observed  Unknown : never evaluated |

| **Skin and Mucosa** | | |
| --- | --- | --- |
| **Field** | **Definition** | **Options** |
| Active Cutaneous Lupus | Butterfly/Malar rash (flat or raised erythematous rash over the cheeks and nasal bridge that typically spares the nasolabial folds).  and/or  Dependent palpable purpura  and/or  Urticarial vasculitis (eruption of erythematous wheals that clinically resemble urticaria)  and/or  Hair loss or non-androgenic effluvium | Past/Present/No/Unk  Past = observed and resolved  Present : at the visit  No : never observed/reported  Unk : not verified |
| Chronic Cutaneous Lupus | Discoid lupus (disc-shaped, erythematous plaques of varying size, and contain areas of follicular hyperkeratoses, which can result in pigmentary change, permanent, depressed scarring, atrophy, and alopecia)  and/or  Lupus panniculitis (Lupus Profundus) (deep indurations or subcutaneous nodules occurring under normal or, less often, involved skin) | Past/Present/No/Unk  Past = observed and resolved  Present : at the visit  No : never observed/reported  Unk : not verified |
| Apthous ulcers | Oral or nasopharyngeal ulcers (recurrent, multiple, small, round, or ovoid ulcers, with circumscribed margins, having yellow or gray floors and are surrounded by erythematous haloes) | Past/Present/No/Unk  Past = observed and resolved  Present : at the visit  No : never observed/reported  Unk : not verified |
| Photosensitivity | Skin rash as a result of unusual reaction to sunlight, by patient history or physician observation | Past/Present /No/Unk  Past = observed and resolved  Present : at the visit  No : never observed/reported  Unk : not verified |
| Calcinosis cutis | Detected either clinically or radiographically.  Calcinosis is defined as palpable, dermal and/or subcutaneous or intramuscular deposits. It is usually located in digits or over large proximal joints or extensor surfaces of distal extremities. | Yes/No/unk  Yes =ever  No : never  Unk : not verified |
| Skin fibrosis | Skin thickening or hardening anywhere but not due to scarring after injury, trauma. | Past/Present/No/unk  Past = observed and resolved  Present : at the visit  No : never observed/reported  Unk : not verified |
| Puffy Fingers | Swollen digits - a diffuse, usually non pitting increase in soft tissue mass of the digits extending beyond the normal confines of the joint capsule. Normal digits are tapered distally with the tissues following the contours of the digital bone and joint structures. Swelling of the digits obliterates these contours. | Past/Present/No/unk  Past = observed and resolved  Present : at the visit  No : never observed/reported  Unk : not verified |
| Telangectasia | Visible macular dilated superficial blood vessels which collapse upon pressure and fill slowly when pressure is released; distinguishable from rapidly filling spider angiomas with central arteriole and from dilated superficial vessels. | Yes/No/unk  Non –receding condition  Yes=ever  No = never  Unk = not verified |
| Subcutaneous nodules | Firm lumps located under the skin | Past/Present/No/unk  Past = observed and resolved  Present : at the visit  No : never observed/reported  Unk : not verified |
| Sicca Syndrome | Dry eye with positive Schirmer's test (<5 mm) *or* reduced tear-film Break-up time (<10 seconds) *or* punctate Rose Bengal and fluorescein staining of the cornea  and/or  Dry mouth *with* abnormal sialometry *or* abnormal salivar scintigraphy *or* abnormal echography  *not due to*  Prolonged use of drugs including antihistamines, antihypertensives, and antidepressants | Yes/No/unk  Non –receding condition  Yes=ever  No = never  Unk : not verified |

| **Heart** | | |
| --- | --- | --- |
| **Field** | **Definition** | **Options** |
| Pericarditis | Evidence of either dry, effusive or fibrinous pericarditis on Echocardiogram or MRI. | Past/Present/No/Unk  Past : observed on the predefined exams and resolved or not verified since an observation of more than 4 weeks  Present : within 4 weeks and not resolved  No : exam done and never observed  Unknown : never performed, irrespective of clinical signs/symptoms |
| Pulmonary hypertension on Echo | Increased Right Ventricular Systolic Pressure (RVSP) on Echo ≥ 35 mmHg assuming a right-atrial pressure = 5 mmHg (alternative definitions: a transtricuspid velocity ≥ 2.74 m/s *or* a transtricuspid gradient = 30 mmHg).  Echocardiography should have been performed within 6 months. | Past/present/No/Unk  Past : present on echo of more than 6 month and resolved or not verified since the observation  Present : observed on echo <6 month  No : echo not showing pulmonary hypertension  Unk : echo not verified |
| Pulmonary arterial hypertension by right-heart catheterization (PAH) | Evidence of precapillary pulmonary arterial hypertension on right-heart catheterization, defined as:  mPAP ≥ 25 mmHg  and  PWP ≤ 15 mmHg | Yes/No/Unk  Yes = ever  No = never  Unk = not verified |
| Hypertension | History of systemic hypertension (either past, actual or under control with medical treatment). | Yes/No/unk  At the visit  Unk : not verified |
| Arrhythmias | Lown grade 3 or higher arrhythmia on 24-hours recording | Past/Present/No/unk  Past : observed on exam and resolved or not verified since an observation of more than 4 weeks  Present : within 4 weeks and not resolved  No : exam done and never observed  Unknown : never performed, irrespective of clinical signs/symptoms |
| Valve lesions | As detected by Echography:  Moderate-to-severe stenosis *or* insufficiency  and/or  Valve vegetations | Yes/No/unk  Non –receding condition  Yes=ever  No = never  Unk : if echo not verified |

| **Lung** | | |
| --- | --- | --- |
| **Field** | **Definition** | **Options** |
| Basilar crackles | As assessed by clinical examination. | Yes/No/unk  At the visit  Unk = not verified |
| Pleural effusion | Evidence of pleural effusion by radiology | Past/Present/No/unk  Past : observed on exam and resolved or not verified since an observation of more than 4 weeks  Present : within 4 weeks and not resolved  No : exam done , never observedand no clinical evidence  Unknown : never performed, irrespective of clinical signs/symptoms |
| Pulmonary fibrosis | Evidence of pulmonary fibrosis *of any extent* on HRCT with any of the following appearances:  -Ground glass attenuation (a)  -Fine intralobular fibrosis (b)  -Honeycombing (microcistic or macrocistic) (b)  a) This alteration is considered reversible, its occurrence has to be demonstrated in a recent (within 12 months) HRCT.  b) These alterations are considered irreversible and can be considered still present even if HRCT has not been performed in the recent past | Yes/No/unk  Yes : if a) shown in a HRCT of less than 12 month or b) shown on HRCT at any time  No : if a) <12 months on HRCT and b) not shown on HRCT  Unk : HRCT not done or a) shown on HRCT > 12 month |
| Functional ventilatory restriction | Presence of a FVC < 70% of the predicted on PFT performed within 6 months | Yes/No/Unk  yes = seen in PFT<6 months no=not shown on PFT < 6 months unk = PFT not done in the last 6 months |
| Worsening lung function | Change in PFT values vs previous tests (within 12 months).  FVC decrease ≥ 10% vs previous  and/or  DLco decrease ≥ 15% vs previous | Yes/No/unk  Unk : PFT not done in the last 12 month or first evaluation |

| **Muscle-Skeletal** | | |
| --- | --- | --- |
| **Field** | **Definition** | **Options** |
| Arthritis | Occurrence of definite clinical synovitis by clinical examination in ≥ 2 peripheral joints lasting at least two weeks | Past/Present/No/unk  Past if observed and resolved  Present : at the visit  No : never observed/reported  Unk : not verified |
| Erosive arthritis | Evidence of erosive arthritis of any degree in any anatomical district as assessed via any radiological mean (conventional radiography, ultrasound, MRI) | Yes/No/unk  Non –receiding condition  Yes=ever  No = never  Unk : if never verified |
| Muscle weakness | Progressive reduced muscle strength starting from proximal muscles | Past/Present/No/unk  Past if observed and resolved  Present : at the visit  No : never observed/reported  Unk : not verified |
| Evidence of inflammatory myopathy | Increased CPK levels ≥ 2 ULN  *and* actual or previous demonstration of  EMG typical alterations  and/or  Biopsy typical alterations excluding any mimics, such as inclusion body myopathy, muscular dystrophy, necrotizing myopathy or enzyme deficiency. | Yes/No/unk  Non –receding condition  Yes=ever  No = never  Unk : if never verified |

| **Kidney** | | |
| --- | --- | --- |
| **Field** | **Definition** | **Options** |
| Abnormal Creatinine | Serum creatinine levels ≥ 20% ULN  or  Increase in serum creatininte levels ≥ 50% vs baseline (also in presence of normal creatinine leves. E.g. baseline = 0.5 mg/dL, actual=0.8 mg/dL)  or  Reduced GFR < 60 mL/min | Past/Present/No /unk  Past : observed and resolved or not verified since an observation of more than 4 weeks  Present : within 4 weeks and not resolved  No : exam done and never observed  Unknown : never evaluated |
| Abnormal urine analysis | haematuria > 2+ on dipstick  or  > 10 red dysmophic blood cells/high-power field  or  cellular casts (granular, tubular or mixed) | Past/Present/No/unk  Past : observed and resolved or not verified since the observation  Present : within 4 weeks and not resolved  No : exam done and never observed Unknown : never evaluated |
| Urine Proteins | Abnormal urine protein creatinine ratio (> 0.2) | Past/Present/No/unk  Past : observed and resolved or not verified since the observation  Present : within 4 weeks and not resolved  No : exam done and never observed Unknown : never evaluated |
| Proteinuria | Excess of serum proteins in the 24-hours urine with the following threshold:  -No (mild): <500 mg/24 hours  -Moderate: 500-3500 mg/24 hours  -Severe (Nephrotic) > 3500 mg/24 hours | No/Moderate/Severe/Past/unk  Past : observed and resolved or not verified since the observation  Moderate/severe : shown in the last 4 weeks  No : exam done and never observed Unk : never evaluated |
| Biopsy proven nephritis | Evidence on biopsy of any type:  -Lupus Nephritis (any class)  -Systemic sclerosis renal disease | Yes/No/ unk  Non –receding condition  Yes=ever  No = never  Unk : if never verified |
| Abnormal lipid profile | Serum tryglicerides > UNL  and  Serum total Cholesterol > UNL  and/or  Serum HDL Cholesterol < LLN | Past/Present/No/unk  Past : observed and resolved or not verified since the observation  Present : within 4 weeks and not resolved  No : exam done and never observed Unknown : never evaluated |

| **Nervous system** | | |
| --- | --- | --- |
| **Field** | **Definition** | **Options** |
| Involvement of the CNS | Presence of any of the following conditions, in absence of infections, metabolic or endocrine disturbances, adverse drug reactions or any other predisposing condition unrelated to systemic autoimmune diseases.  -Transitory Ischaemic Attacks  -Ischaemic stroke  -Mild-to-moderate cognitive disfunction with impaired attention or visual memory or verbal memory or impaired executive function and psychomotor speed.  -Seizures  -Chorea  -Acute Confusional State characterized by acute onset, fluctuating level of consciousness with decreased attention  -Psychiatric Disorders characterized by delusions (false beliefs refuted by objective evidence) or hallucinations (perceptions in the absence of external stimuli).  -Transverse myelitis  -Aseptic meningitis  -Inflammatory optic neuritis  -Ischaemic/thrombotic optic neuropathy | Past/Present/No/unk  Past if observed and resolved  Present : at the visit  No : never observed/reported  Unk : not verified |
| Involvement of the PNS | EMG demonstration of neuropathy (polyneuropathy, mononeuropathy) in absence of CNS involvement excluded by neuroimaging. | Past/Present/No/unk  Past if observed and resolved  Present : at the visit  No : never observed/reported  Unk : not verified |

| **Concomitant Therapy** | | |
| --- | --- | --- |
| **Field** | **Definition** | **Options** |
| Current use of Antimalarials | Current use (= within 4 weeks) | Yes/No |
| Current use of systemic antibiotics | Current use (= within 4 weeks) | Yes/no |
| Current use of Immunosoppressants | Current use (= within 4 weeks) | Yes/no |
| Steroids | Current use of steroids.  When Yes is selected the average weekly dose (last week), expressed in prednisone mg/day or equivalent should be specified in the entry box. | Yes/No + Blank |
| Biologicals | No  Anti-TNF (any molecule)  Tocilizumab (or other anti IL6)  Abatacept | Scroll-Down |

| **Comorbidities** | |
| --- | --- |
| **Field** | **Options** |
| Abdominal pain | Yes/No/unk |
| Arrhythmia* | Yes/No/unk |
| Asthma | Yes/No/unk |
| Atopic dermatitis | Yes/No/unk |
| Autoimmune Cholangitis | Yes/No/unk |
| Chronic Obstructive Pulmonary Disease (COPD) | Yes/No/unk |
| Chronic Infections | Yes/No/unk |
| Cigarette Smoking | Yes/No/unk |
| Coeliac disease | Yes/No/unk |
| Coronary artery disease | Yes/No/unk |
| Diabetes | Yes/No/unk |
| Diarrhea (recurrent) | Yes/No/unk |
| Dyslipidemia | Yes/No/unk |
| History of Cancer | Yes/No/unk |
| Hypertension* | Yes/No/unk |
| Inflammatory Bowel Disease | Yes/No/unk |
| Inherited Anemia | Yes/No/unk |
| Obesity (BMI ≥ 30) | Yes/No/unk |
| Primary Biliary Cirrhosis | Yes/No/unk |
| Psoriasis | Yes/No/unk |
| Reflux disease* | Yes/No/unk |
| Statin use | Yes/No/unk |
| Steroid use* | Yes/No/unk |
| Stipsis /constipation | Yes/No/unk |
| Thyroiditis | Yes/No/unk |
|  |  |
| *Fields to be collected in Healthy controls only (already included in the patients’ items) | |

*For receding conditions yes refer to the current status*

**List of abbreviations**

ALP, ALkaline Phosphatase

ALT, ALanine aminoTransferase

AST, ASpartate aminoTransferase

BMI, Body Mass Index

CNS, Central Nervous System

CPK, Creatine PhosphoKinase

CRP, C-Reactive Protein

DLco, Diffusing Lung capacity for Carbon Monoxyde

EMG, needle ElectroMyoGraphy

ESR, Erythrocite Sedimentation Rate

FVC, Forced Vital Capacity

GFR, Glomerular Filtration Rate

gGT, Gamma Glutamyl Transferase

Hb, Hemoglobin

HDL, High-Density Lipoprotein

HRCT, High Resolution Computed Tomography

MRI, Magnetic Resonance Imaging

mPAP, mean Pulmonary Artery Pressure

PFT, Pulmonary Function Tests

PLT, PLaTelets

PNS, Peripheral Nervous System

PWP, Pulmonary Wedge Pressure

LLN, Lower Laboratory Normal

ULN, Upper Laboratory Normal

WBC, White Blood Cells
