# Supplementary material for: An elevated polyclonal free light chain level reflects a strong interferon signature in patients with systemic autoimmune diseases
Source: J Transl Autoimmun. 2021 Mar 2;4:100090. doi: 10.1016/j.jtauto.2021.100090 (PMC8010703; doi:10.1016/j.jtauto.2021.100090)
Supplement: Multimedia component 3 [file mmc3.pptx]

## Slide 1
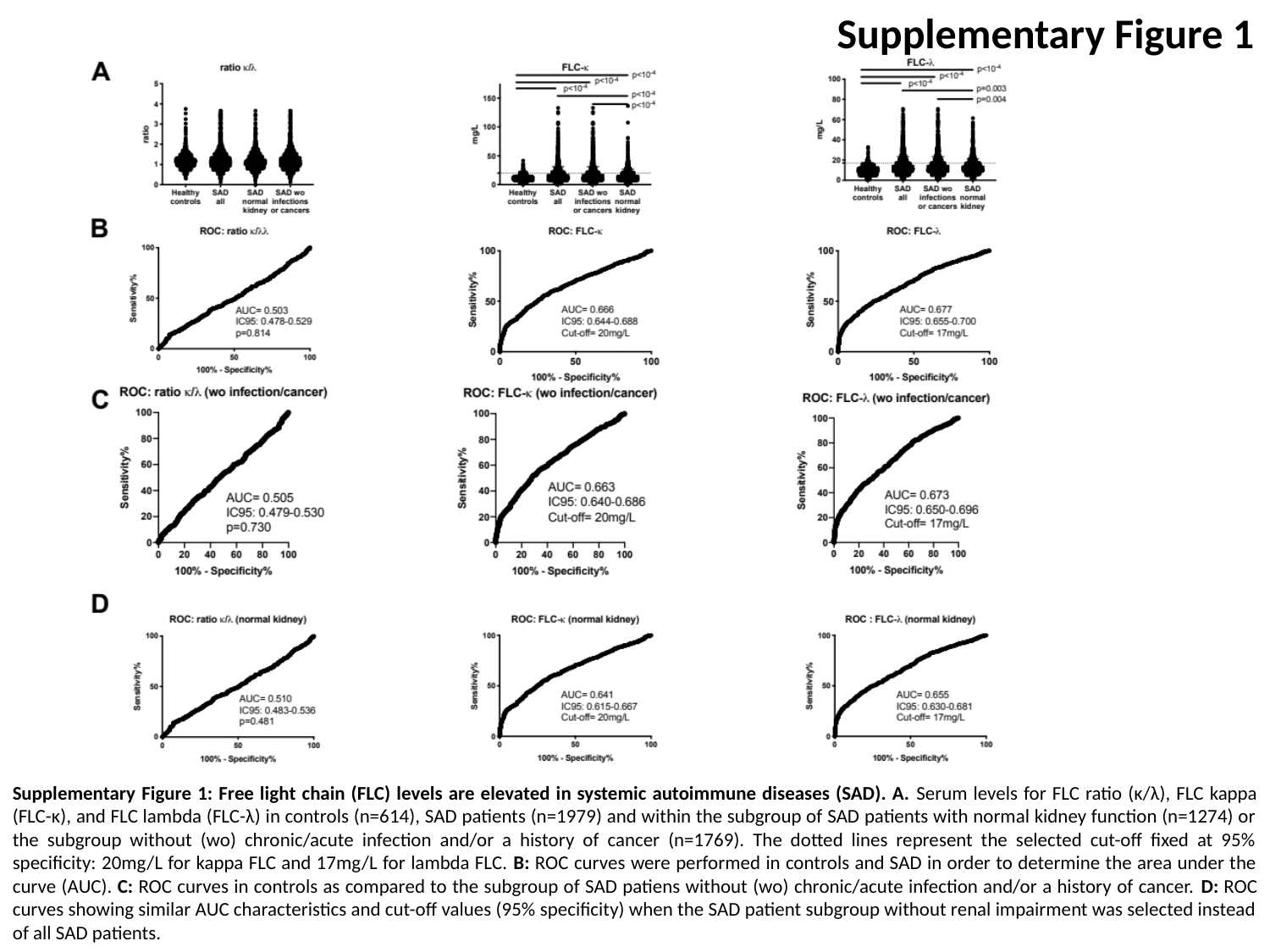

Supplementary Figure 1
Supplementary Figure 1: Free light chain (FLC) levels are elevated in systemic autoimmune diseases (SAD). A. Serum levels for FLC ratio (κ/λ), FLC kappa (FLC-κ), and FLC lambda (FLC-λ) in controls (n=614), SAD patients (n=1979) and within the subgroup of SAD patients with normal kidney function (n=1274) or the subgroup without (wo) chronic/acute infection and/or a history of cancer (n=1769). The dotted lines represent the selected cut-off fixed at 95% specificity: 20mg/L for kappa FLC and 17mg/L for lambda FLC. B: ROC curves were performed in controls and SAD in order to determine the area under the curve (AUC). C: ROC curves in controls as compared to the subgroup of SAD patiens without (wo) chronic/acute infection and/or a history of cancer. D: ROC curves showing similar AUC characteristics and cut-off values (95% specificity) when the SAD patient subgroup without renal impairment was selected instead of all SAD patients.

## Slide 2
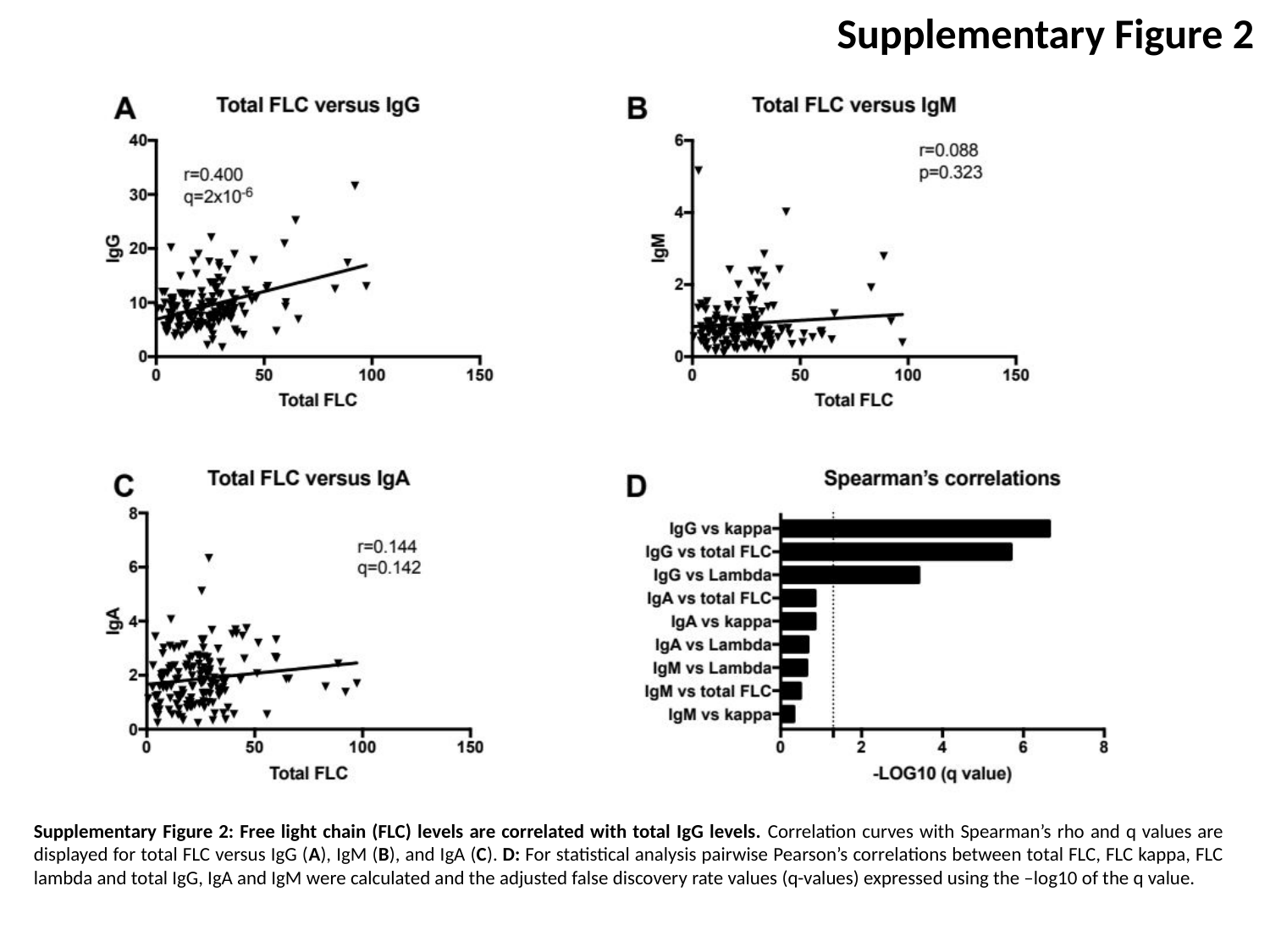

Supplementary Figure 2
Supplementary Figure 2: Free light chain (FLC) levels are correlated with total IgG levels. Correlation curves with Spearman’s rho and q values are displayed for total FLC versus IgG (A), IgM (B), and IgA (C). D: For statistical analysis pairwise Pearson’s correlations between total FLC, FLC kappa, FLC lambda and total IgG, IgA and IgM were calculated and the adjusted false discovery rate values (q-values) expressed using the –log10 of the q value.

## Slide 3
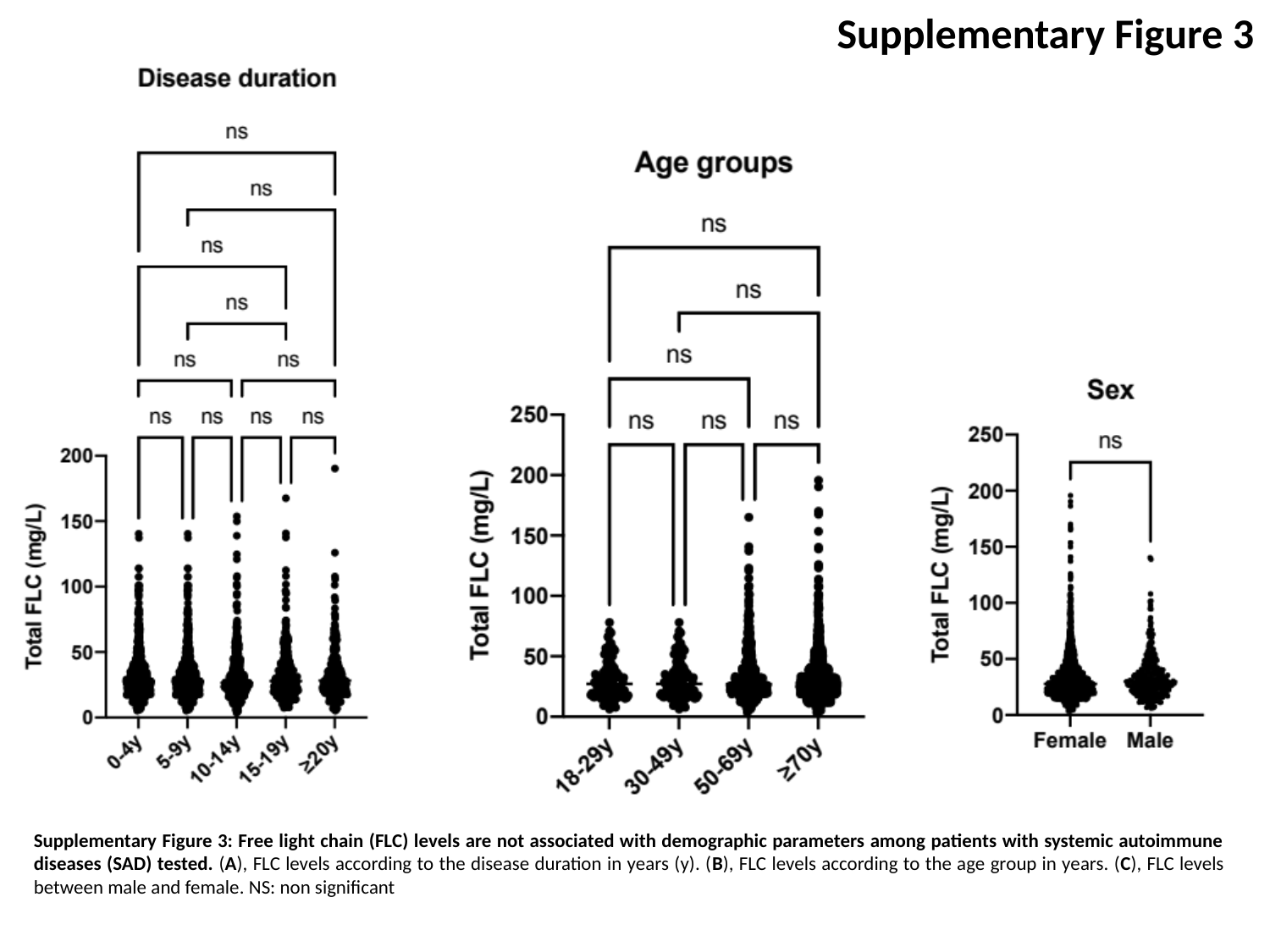

Supplementary Figure 3
Supplementary Figure 3: Free light chain (FLC) levels are not associated with demographic parameters among patients with systemic autoimmune diseases (SAD) tested. (A), FLC levels according to the disease duration in years (y). (B), FLC levels according to the age group in years. (C), FLC levels between male and female. NS: non significant
